# Supplementary material for: Tissue Specific Dual RNA-Seq Defines Host–Parasite Interplay in Murine Visceral Leishmaniasis Caused by Leishmania donovani and Leishmania infantum
Source: Microbiol Spectr. 2022 Apr 6;10(2):e00679-22. doi: 10.1128/spectrum.00679-22 (PMC9045295; doi:10.1128/spectrum.00679-22)
Supplement: SUPPLEMENTAL FILE 7 — Supplemental material. Download SPECTRUM00679-22_Supp_7_seq14.pdf, PDF file, 0.2 MB [file spectrum00679-22_supp_7_seq14.pdf]

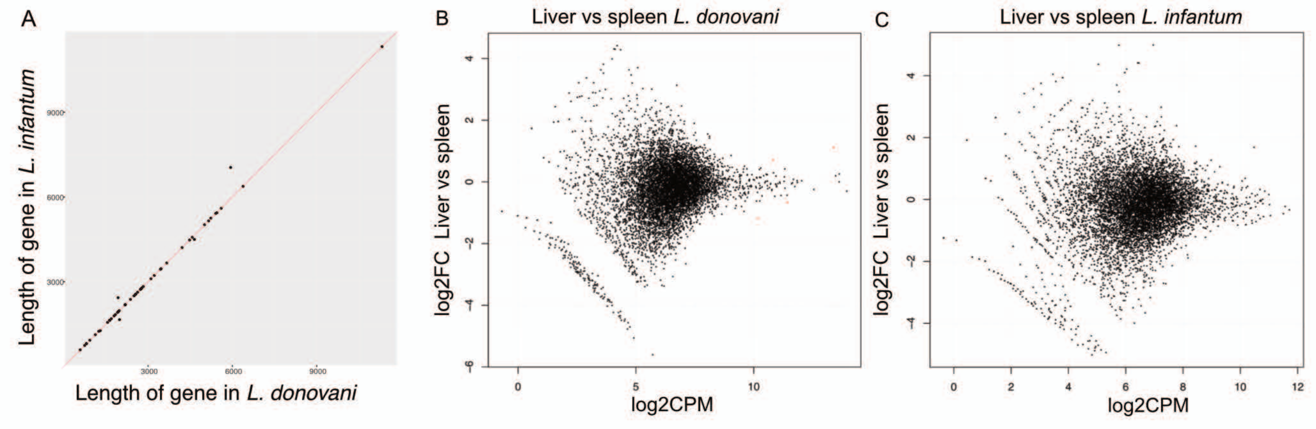
A
Length of gene in L. infantum
Length of gene in L. donovani
3000
6000
9000
B
Liver vs spleen L. donovani
log2FC Liver vs spleen
log2CPM
C
Liver vs spleen L. infantum
log2FC Liver vs spleen
log2CPM
