# Supplementary figures and images for: Tissue Specific Dual RNA-Seq Defines Host–Parasite Interplay in Murine Visceral Leishmaniasis Caused by Leishmania donovani and Leishmania infantum
Source: Microbiol Spectr. 2022 Apr 6;10(2):e00679-22. doi: 10.1128/spectrum.00679-22 (PMC9045295; doi:10.1128/spectrum.00679-22)

Spleen

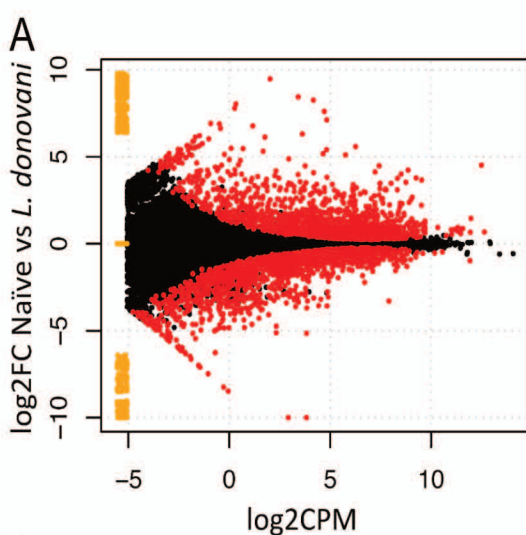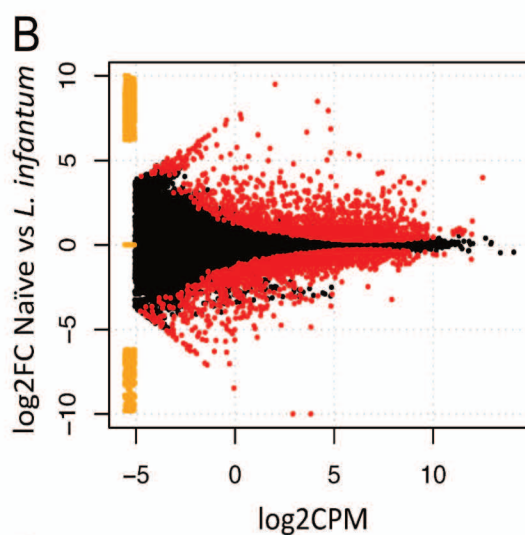

Liver

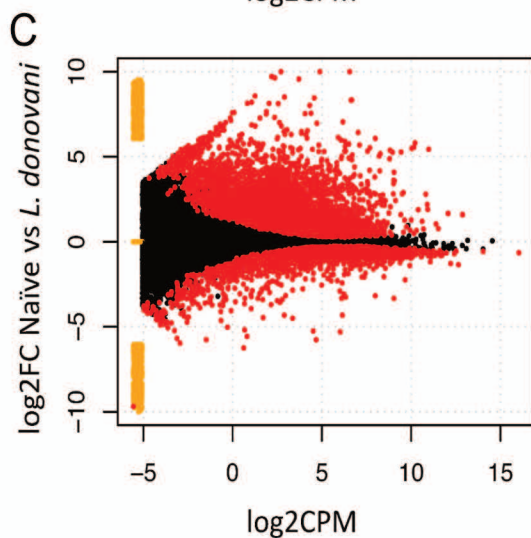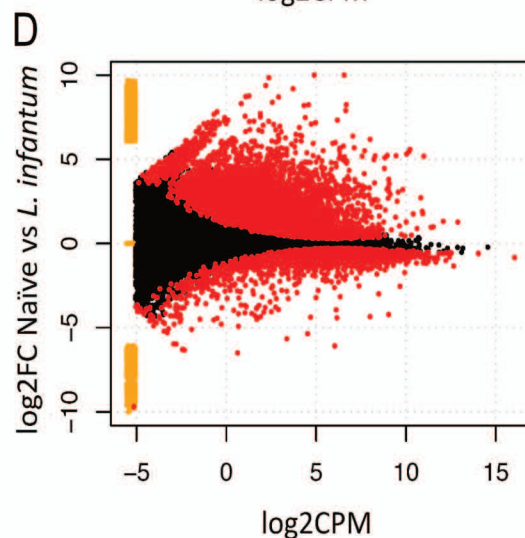log2FC Uninfected vs *L. infantum*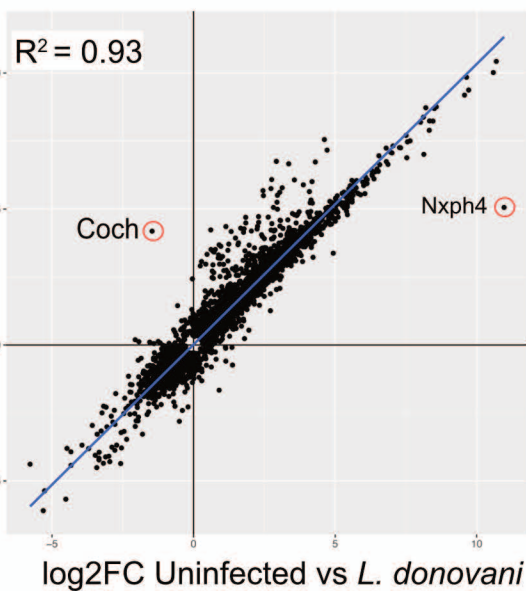log2FC Uninfected vs *L. infantum*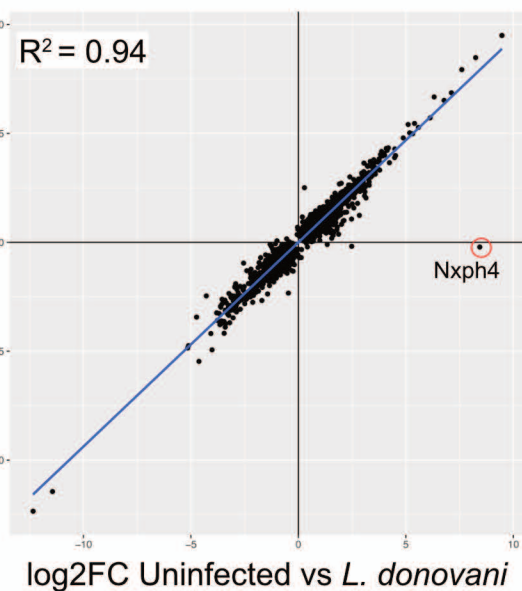

Supplement: SUPPLEMENTAL FILE 8 — Supplemental material. Download SPECTRUM00679-22_Supp_8_seq15.pdf, PDF file, 0.5 MB [file spectrum00679-22_supp_8_seq15.pdf]

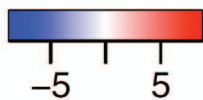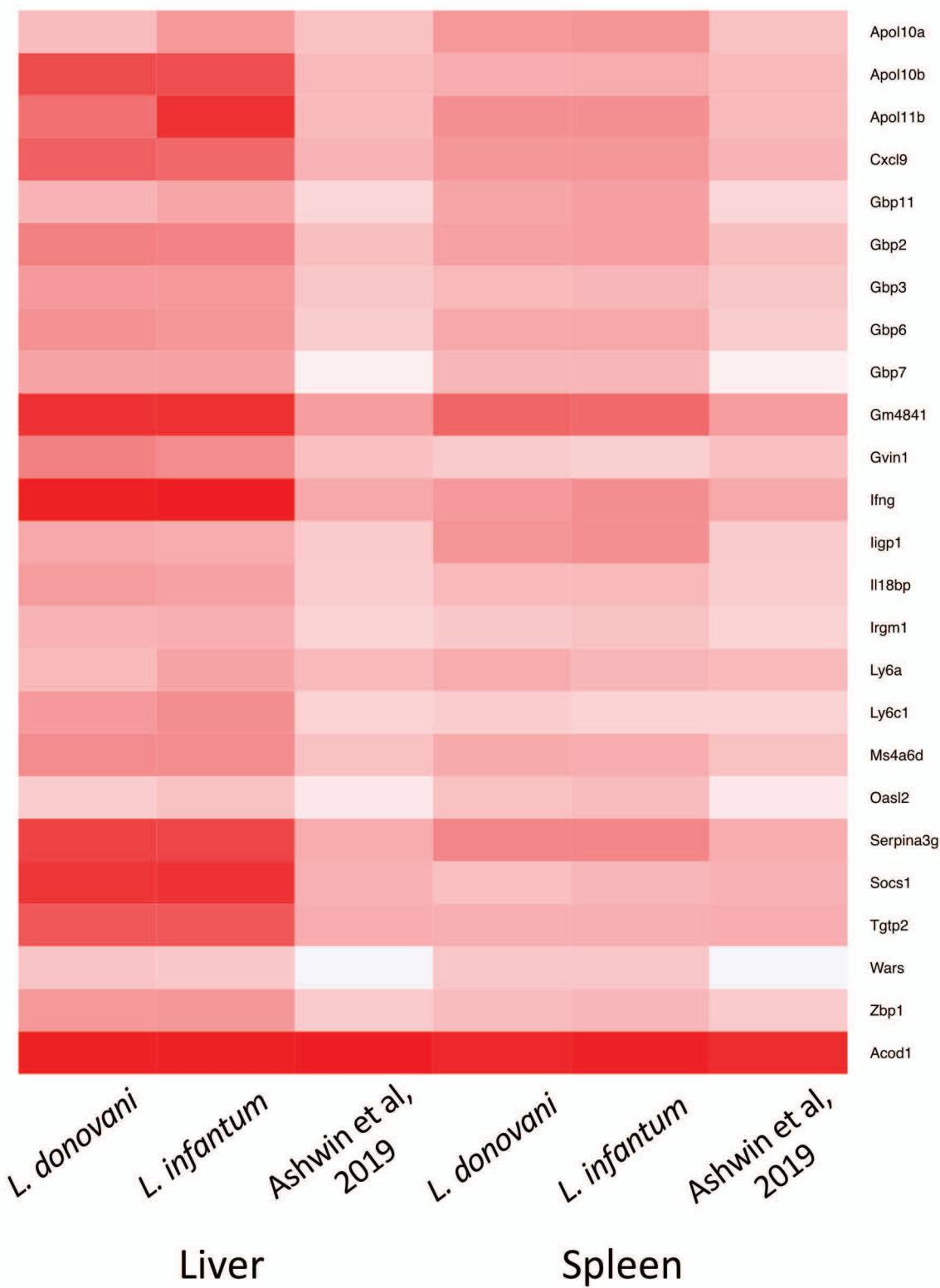

Supplement: SUPPLEMENTAL FILE 10 — Supplemental material. Download SPECTRUM00679-22_Supp_10_seq17.pdf, PDF file, 0.2 MB [file spectrum00679-22_supp_10_seq17.pdf]
