# Supplementary material for: Tissue Specific Dual RNA-Seq Defines Host–Parasite Interplay in Murine Visceral Leishmaniasis Caused by Leishmania donovani and Leishmania infantum
Source: Microbiol Spectr. 2022 Apr 6;10(2):e00679-22. doi: 10.1128/spectrum.00679-22 (PMC9045295; doi:10.1128/spectrum.00679-22)
Supplement: SUPPLEMENTAL FILE 9 — Supplemental material. Download SPECTRUM00679-22_Supp_9_seq16.pdf, PDF file, 0.3 MB [file spectrum00679-22_supp_9_seq16.pdf]

A. Liver

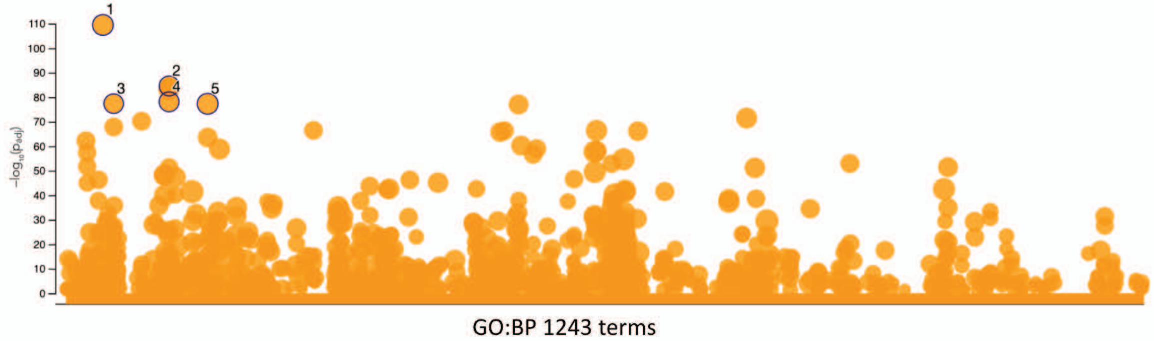

B. Annotated top enriched terms in the liver

ID	Source	Term ID	Term Name	p_{adj} (query_1)
1	GO:BP	GO:0002376	immune system process	3.458×10^{-110}
2	GO:BP	GO:0006955	immune response	2.634×10^{-85}
3	GO:BP	GO:0002682	regulation of immune system process	4.298×10^{-78}
4	GO:BP	GO:0006952	defense response	7.734×10^{-79}
5	GO:BP	GO:0009605	response to external stimulus	4.963×10^{-78}

C. Spleen

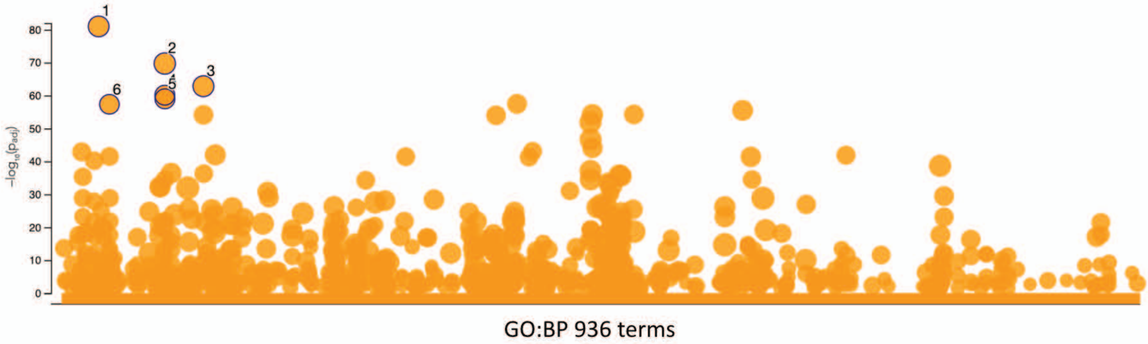

D. Annotated top enriched terms in the spleen

ID	Source	Term ID	Term Name	p_{adj} (query_1)
1	GO:BP	GO:0002376	immune system process	1.034×10^{-81}
2	GO:BP	GO:0006950	response to stress	1.927×10^{-70}
3	GO:BP	GO:0009605	response to external stimulus	1.501×10^{-63}
4	GO:BP	GO:0006952	defense response	8.116×10^{-61}
5	GO:BP	GO:0006955	immune response	9.792×10^{-60}
6	GO:BP	GO:0002682	regulation of immune system process	4.685×10^{-58}
